# Development of novel methodology for the molecular differentiation of *Cryptosporidium parvum gp60* subtypes via high resolution melting analysis

**DOI:** 10.1016/j.mex.2020.101157

**Published:** 2020-11-26

**Authors:** Jennifer K. O’ Leary, Liam Blake, Gerard D. Corcoran, Roy D. Sleator, Brigid Lucey

**Affiliations:** a Department of Biological Sciences, Cork Institute of Technology, Bishopstown, Cork, Ireland; b Department of Clinical Microbiology, Cork University Hospital, Wilton, Cork, Ireland

**Keywords:** Molecular epidemiology, *Cryptosporidium*, Enteric parasitology, DNA sequencing, Real-time PCR, High resolution melting(HRM) analysis, *gp60* gene, Infectious disease

## Abstract

*Cryptosporidium* species subtypes are generally identified *via* DNA sequencing of the *gp60* gene tandem repeat motif region. Due to the immunogenic nature of its glycoprotein products, *gp60* is subject to host selective pressures, genetic recombination and evolutionary processes that drive extensive polymorphism at this locus. The elucidation of the polymorphic nature of this gene has led to the current mainstay in *Cryptosporidium* subtyping nomenclature.

This study aimed to develop a real-time polymerase chain reaction based method utilising a post-PCR application, high resolution melting (HRM) analysis, in conjunction with the abovementioned *gp60* nomenclature system, in order to differentiate between *Cryptosporidium parvum gp60* subtypes. Subtype differentiation is based on the difference between the melting temperatures of individual subtypes conferred by variations in the polymorphic region of *gp60*.

• Nested *gp60* primers were designed to amplify a target region of <200 base pairs for effective HRM analysis

• This method presents a rapid, sensitive, cost effective alternative to conventional sequencing.

• This method is highly flexible and may be applied to other loci in order to facilitate multi-locus analysis and improve the discriminative abilities of the method.

## Graphical abstract

**Figure:**
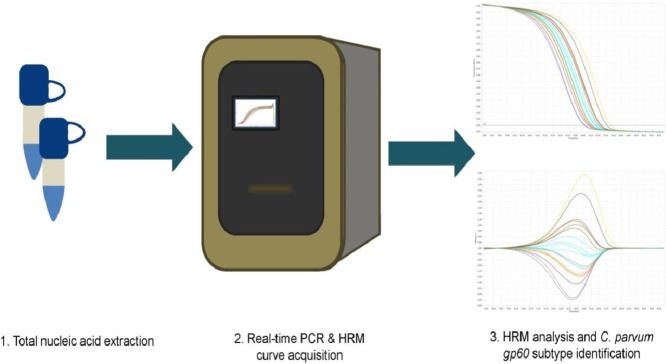

Specifications table

**Table:** 

Subject Area:	Immunology and Microbiology
More specific subject area:	Molecular Parasitology
Method name:	60 kDa glycoprotein gene based differentiation of *Cryptosporidium parvum* subtype isolates *via* high resolution melting (HRM) analysis.
Name and reference of original method:	N/A
Resource availability:	N/A

## Method details

### Method background

Gastrointestinal and diarrhoeal disease contribute significantly to global morbidity and mortality, accounting for more than 1.3 million deaths in 2015 [1]. *Cryptosporidium*, a globally ubiquitous, protozoan parasite, is an aetiology to which diarrhoea induced death is commonly attributed. Over 90% of cases of cryptosporidiosis are attributable to two species, *Cryptosporidium parvum* and *Cryptosporidium hominis*; although nearly 20 species and genotypes have been reported in humans [2]. First reported as an agent of human disease in the 1976 [[3],[4]], cryptosporidiosis, in addition to disproportionately affecting immunocompetent children under the age of five, can result in protracted or recurrent bouts of potentially fatal diarrhoeal illness in immunocompromised individuals.

Previously, diagnostic analysis of this enteric pathogen was predominantly limited to microscopy *via* fluorescent or acid-fast staining. However, microscopic analysis of oocysts precluded thorough epidemiological analyses, lacking in the ability to distinguish between species of *Cryptosporidium* morphologically. However, since the release of both the *C. parvum* and *C. hominis* genomes in the early 2000s [5], [6], [7], a host of molecular methods have been developed to distinguish between *Cryptosporidium* species and subspecies, with species identification largely achieved through polymerase chain reaction-restriction fragment length polymorphism (PCR-RFLP) of various genes and DNA sequencing or real-time PCR based analysis of the small subunit ribosomal RNA (SSU rRNA or 18S), a method which has become the mainstay in differentiating *C. parvum* and *C. hominis* [8], [9], [10], [11]. Subtype identification is conducted through DNA sequence analysis of the 60 kDa glycoprotein gene (*gp60*) in accordance with nomenclature developed by Strong et al. and modified by Sulaiman et al. [12,13]. Within this system, subtype assignment is based on the number of TCA repeats (represented by the letter A), TCG repeats (represented by the letter G), TCT repeats (represented by the letter T) and other repetitive sequences, such as the ACATCA (represented by the letter R) associated with the *C. parvum* IIa allele family, within the *gp60* tandem repeat motif region. Subtype families are named as Ia, Ib, Ic, Id, Ie, If, etc. for *C. hominis* and IIa, IIb, IIc, IId, etc. for *C. parvum*, with further species families named in ascending order.

The ability to analyse *Cryptosporidium* species and subtypes is critical as both inter- and intra-species variation in host range and/or virulence/transmissibility have been described [2]. Additionally, *Cryptosporidium* species exhibit regional variation; with both *C. parvum* and *C. hominis* predominance found to vary between industrialised countries, while *C. hominis* tends to predominate in developing countries [14]. It is worth noting that *C. parvum* is the predominant species in several European countires [15]. Within *Cryptosporidium* species, *gp60* subtypes also exhibit regional variation, with *C. parvum* IIa subtypes the most prevalent case of infection in industrialised nations [16]. Indeed, *gp60* is one of the most polymorphic genes in the *Cryptosporidium* genome, subject to selective pressures and genetic recombination. Consequently, *Cryptosporidium* populations are often panmictic in structure, with this structure particularly common within *C. parvum* [17,18]. Thus, analysis of the regional variation exhibited by various *Cryptosporidium gp60* subspecies is also key to understanding and elucidating the population genetics.

The development of real-time PCR, a rapid, highly sensitive, fluorescence based technology has revolutionised clinical microbiology, with myriad potential applications in molecular parasitology. Real-time PCR also boasts a suite of advanced post-PCR applications, one of which, high resolution melting (HRM) analysis, is capable of interrogating DNA sequence variations *via* determination of the relationship between melting temperature and DNA fragment denaturation [19]. HRM analysis is sensitive enough to resolve a single base pair difference between amplicons, and has been applied to single nucleotide polymorphism (SNP) and tandem-repeat number based genotyping and determination of DNA methylation status [19]. Recently HRM analysis has been applied to the differentiation of *Cryptosporidium* species from other apicomplexan parasites, *Toxoplasma gondii, Sarcocystis* species, *Neospora* species, and to differentiate between *Cryptosporidium* species [20], [21], [22]. HRM analysis has also been successfully applied to the intra-species differentiation of *Cryptosporidium cuniculus gp60* subtypes Va and Vb [23].

In this study, following confirmation of isolate bank species and subtype designations *via* a real-time PCR based 18S rRNA gene speciation method and DNA sequencing of the *gp60* gene, respectively, HRM analysis was applied to the differentiation of *C. parvum gp60* subtypes of allele family IIa. This subtype family is responsible for the majority of *C. parvum* cases in Europe [15]. Additionally, it has been established that analysis of multiple loci is required for reliable and sufficiently discriminatory genotyping of *Cryptosporidium* subtypes [24]. This study was conducted in order to develop a HRM analysis based method capable of rapid, detailed epidemiological analyses using the locus of interest, that was also capable of being expanded upon for future development of a multi-locus variable number tandem repeat analysis (MLVA) tool. Such a tool would improve the discriminatory power of the method and would also be applicable to any future pan- European multi-locus genotyping schemes that may be introduced.

## Ethical statement

Full ethical approval was obtained from the Cork Institute of Technology (CIT) Research Ethics Committee prior to study commencement (reference number MF-C-JOL12/2014).

### Clinical sample acquisition and total nucleic acid extraction

Samples were acquired through collaboration with the Clinical Microbiology Department of Cork University Hospital (CUH). Over a period of three years, from August 2015 to August 2018, inclusive, 163 *Cryptosporidium* positive isolates were amassed during routine molecular enteric pathogen screening. Sample acceptance for routine molecular enteric pathogen screening was limited to samples from patients presenting with diarrhoea, with an acceptance criterion requiring samples to be graded as type 5 or higher on the Bristol Stool Form Chart (BSFC).

The EntericBio GastroPanel II (Serosep, Limerick, Ireland) one-step, heat treatment extraction protocol, a preliminary stage in the routine molecular enteric pathogen screening process, was utilised, as per manufacturer instructions, for sample DNA extraction.

• EntericBio Stool Preparation Solution (SPS) was inoculated with a sample coated FloqSwab (Copan, Italy) .
• The sample inoculated SPS tube was vortexed for 30 s.
• The inoculated SPS tube was then heated at 103 °C for 30 min to achieve DNA extraction.

### 18S rRNA gene based species identification

Clinical enteric pathogen screening was limited to genus level identification. Consequently, a previously published method by Mary et al., employing real-time polymerase chain reaction (PCR) based amplification of an 18S rRNA gene target, was used to rapidly differentiate between *C. parvum* and *Cryptosporidium hominis* isolates [25]. This method, as described by Mary et al. [25], employed a pan-*Cryptosporidium* specific primer pair directed at a conserved region of the18S rRNA gene, while two varying minor groove binding fluorescent probes differentiated between *C. parvum* and *C. hominis*, respectively. The following modifications to the method were performed: the VIC fluorescent dye bound to the minor groove binding probe for *C. parvum* was replaced with HEX, in order to align with the LightCycler96 (LC96) (Roche Molecular Diagnostics, Germany) detection specifications; on-site optimisation of the method on the LC96 instrument indicated optimal probe concentrations to be 50 nM, rather than the 100 nM as specified by Mary et al. [25].

Multiplex real-time PCR step-wise description:

• All reactions were carried out at a final volume of 20 µl, with mastermix constituent volumes and concentrations outlined in Table 1. These volumes were multiplied by the desired number of reactions plus an additional 10%, to account for pipetting error.

**Table 1:** Multiplex 18S rRNA gene real-time PCR mastermix components.

Reagent	Volume (μl)	Final Concentration
LightCycler 480 High Resolution Melting Master	4	1x
Molecular grade water	10	N/A
Forward primer (10 µM) (‘5 – CATGGATAACCGTGGTAAT – 3′)	0.4	200 nM
Reverse primer (10 µM) (‘5 –TACCCTACCGTCTAAAGCTG,– 3′)	0.4	200 nM
*C. parvum* probe (10 µM) (HEX-ATCACATTAAATGT-MGBBHQ)	0.1	50 nM
*C. hominis* probe (10 µM) (FAM-ATCACAATTAATGT-MGB-BHQ)	0.1	50 nM
Template	5	N/A
• 15 µl of the prepared mastermix was added to each reaction tube.
• Lastly, 5 µl of genomic template DNA was added to the relevant reaction tube.
• Real-time PCR reactions were conducted on a Roche LC96 thermocycler under the following cycling conditions: initial denaturation at 94 °C for 10 min, subsequent 3-step amplification for 45 cycles, including denaturation at 94 °C for 10 s, annealing at 54 °C for 30 s and extension at 72 °C for 10 s.

Additional notes:

• The concentration of genomic *Cryptosporidium* DNA present in each sample could not be determined, as any quantified value would represent the total nucleic acid concentration derived from all microorganisms present in the original faecal sample, given the general extraction protocol employed.

### *gp60* primer design

MUSCLE software (https://www.ebi.ac.uk/Tools/msa/muscle/) was utilised to conduct multiple sequence alignment on subtype sequences from *C. parvum gp60* allele families prevalent in Europe [15], specifically the IIa-IIj families (GenBank accession numbers: AB242224-AB242229, AY382675, AY738185, AY738186, AY738188-AY7381889, AY873780-AY873782, AY738191, AY738195, DQ192502- DQ192508, DQ630514-DQ630515, DQ630517, DQ630519, DQ648531-DQ648537, EU140508), in order to identify homologous regions circumscribing the tandem repeat region of *gp60* between the various subspecies.

The online primer designing tool, Primer-Blast (www.ncbi.nlm.nih.gov/tools/primer-blast/), was used to design both outer and inner primers within these homologous regions. The characteristics of the resultant primers are outlined in Table 2. Due to the presence of subtype dependant variation in the number of tandem repeats present in the targeted region, amplicon sizes vary slightly.

**Table 2:** *gp60* gene targeting primer pairs designed for use in *gp60* subtyping and HRM analysis.

Primer name	Target gene	Annealing temperature (°C)	Sequence (5′ – 3′)	Fragment size (bp)
gp60Outer	60 kDa glycoprotein gene	65	F: TCTCCGTTATAGTCTCCGCTGT R: TGCGGGATCTGTTTGGTCTT	462 - 498
gp60Inner	60 kDa glycoprotein gene	60	F: CCTTCCGTTATAGTCTCCGCT R: CTTCTCCGCCATCTGCTTCT	141 – 177

### First round real-time PCR, amplicon purification and sample sequencing

All first round real-time PCR amplifications, employing the gp60Outer primer pair, were conducted using the LC96 instrument (Roche, Basel, Switzerland). The reagent volumes necessary for a single reaction are outlined in Table 3. These volumes were multiplied by the desired number of reactions plus an additional 10%, to account for pipetting error.

**Table 3:** First round *gp60* real-time PCR mastermix components.

Reagent	Volume (μl)	Final Concentration
FastStart Essential DNA Green Master (Roche, Basel, Switzerland)	10	1x
Molecular grade water	4.2	N/A
Gp60Outer forward primer (10 µM)	0.4	200 nM
Gp60Outer reverse primer (10 µM)	0.4	200 nM
Template	5	N/A

Step-wise description:

• During reaction mastermix preparation, DNA was withheld from the mastermix.
• Reactions were conducted at a volume of 20 µl.
• 15 µl of the prepared mastermix was added to each reaction tube.
• 5 µl of genomic template DNA was subsequently added to the relevant reaction tube.
• Real-time PCR reactions were conducted under the following cycling conditions: initial denaturation at 95 °C for 10 min, subsequent 3-step amplification for 45 cycles, including denaturation at 95 °C for 30 s, annealing at 65 °C for 30 s and extension at 72 °C for 40 s.
• All resulting clinical isolate amplicons were purified using the High Pure PCR product purification kit (Roche Molecular Diagnostics, Germany).
• Samples were sequenced bidirectionally off-site via Sanger sequencing (Eurofins, Cologne, Germany).

Additional notes:

• Amplicon purification was conducted as per the manufacturer's instructions, with one minor modification. At the elution phase of the purification protocol, 20 µl elution buffer was added to the column, instead of the indicated 50 µl, and an additional incubation step at 35 °C for 5 min was included prior to centrifugation, in order to improve DNA yield.

### *gp60* subtype identification

Sequence data were subsequently analysed and *gp60* subtype designations were successfully determined for 149 of the 163 samples (91.4%).

Overall, 12 *C. parvum* subtypes were identified amongst the clinical samples, with one of each sample of each subtype selected for further analysis. An additional 6 reference samples were also included in the study, giving a total of 18 *C. parvum* family IIA subtypes selected for HRM analysis, as outlined in Table 4.

**Table 4:** Provenance of *gp60* subtyped *C. parvum* isolates included in the HRM analysis study.

Species	gp60 Subspecies Designation	Source	Country of origin
*C. parvum*	IIaA10G2R1	Clinical Isolate	Ireland
*C. parvum*	IIaA15G1R2	Clinical Isolate	Ireland
*C. parvum*	IIaA15G2R1	Clinical Isolate	Ireland
*C. parvum*	IIaA16R1	Reference Sample	United Kingdom
*C. parvum*	IIaA16G1R1	Reference Sample	United Kingdom
*C. parvum*	IIaA17G1R1	Clinical Isolate	Ireland
*C. parvum*	IIaA17G2R1	Clinical Isolate	Ireland
*C. parvum*	IIaA17G3R1	Clinical Isolate	Ireland
*C. parvum*	IIaA17G4R1	Clinical Isolate	Ireland
*C. parvum*	IIaA18G1R1	Reference Sample	United Kingdom
*C. parvum*	IIaA18G3R1	Clinical Isolate	Ireland
*C. parvum*	IIaA19G3R1	Clinical Isolate	Ireland
*C. parvum*	IIaA19G4R1	Clinical Isolate	Ireland
*C. parvum*	IIaA20G1R1	Reference Sample	United Kingdom
*C. parvum*	IIaA20G2R1	Reference Sample	United Kingdom
*C. parvum*	IIaA20G3R1	Clinical Isolate	Ireland
*C. parvum*	IIaA21G1R1	Reference Sample	United Kingdom
*C. parvum*	IIaA21G3R1	Clinical Isolate	Ireland

### Nested real-time PCR and HRM curve acquisition

Second round, inner *gp60* primer amplification reactions were conducted on the LC96 analyser (Roche Molecular Diagnostics, Germany). The reagent volumes necessary for a single reaction are outlined in Table 5. These volumes were multiplied by the desired number of reactions plus an additional 10%, to account for pipetting error.

**Table 5:** Second round *gp60* real-time PCR mastermix components.

Reagent	Volume (μl)	Final Concentration
LightCycler 480 High Resolution Melting Master (Roche)	10	1x
Molecular grade water	4.4	N/A
Gp60Inner forward primer (10 µM)	0.6	300 nM
Gp60Inner reverse primer (10 µM)	0.6	300 nM
MgCl_2_ (25 mM)	2.4	3.0 mM
Template	2	N/A

Step-wise description:

• First round reaction PCR products were diluted 1:100 in molecular grade water. During reaction mastermix preparation, DNA was withheld from the mastermix.
• Reactions were conducted at a volume of 20 µl.
• 18 µl of the prepared mastermix was added to each reaction tube.
• 2 µl of genomic template DNA was subsequently added to the relevant reaction tube.
• Real-time PCR reactions were conducted under the following cycling conditions: initial denaturation at 95 °C for 10 min, subsequent 3-step amplification for 35 cycles, including denaturation at 95 °C for 30 s, annealing at 60 °C for 30 s and extension at 72 °C for 40 s.
• Incorporated into the second round real-time PCR cycling conditions, HRM was conducted immediately post-PCR amplification. HRM conditions were as follows: DNA was initially denatured by heating at 95 °C for 60 s, followed by cooling at 40 °C for 60 s and subsequently increasing the temperature by 2.2 °C/s from 65 °C to 97 °C, taking 15 continuous readings/ °C, in order to monitor the change in fluorescence.

Additional notes:

• MgCl_2_ was added as a separate mastermix constituent for second round amplification reactions. LightCycler 480 High Resolution Melting Master Mix(Roche, Basel, Switzerland) does not contain MgCl_2_ to allow for optimisation of MgCl_2_ concentrations, ensuring specific amplification for optimal HRM analysis.

### High Resolution Melting (HRM) analysis of inner *gp60* amplicons

Following high resolution melting-curve acquisition, the resulting data were analysed and a normalisation region of 79 to 86 °C was applied for analysis within the LC96 software (Roche Molecular Diagnostics, Basel, Switzerland). The positive/negative threshold was set to the default 0.05 relative fluorescence units (RFU). Delta melting temperature (T_m_) discrimination and curve shape discrimination parameters were set to 100%. Normalised melting curve, differential melting peak, and difference plots were generated and analysed in order to determine the precise melting temperature of each *C. parvum* subtype. Analysis was conducted on all duplicate runs.

**Fig. 1:**
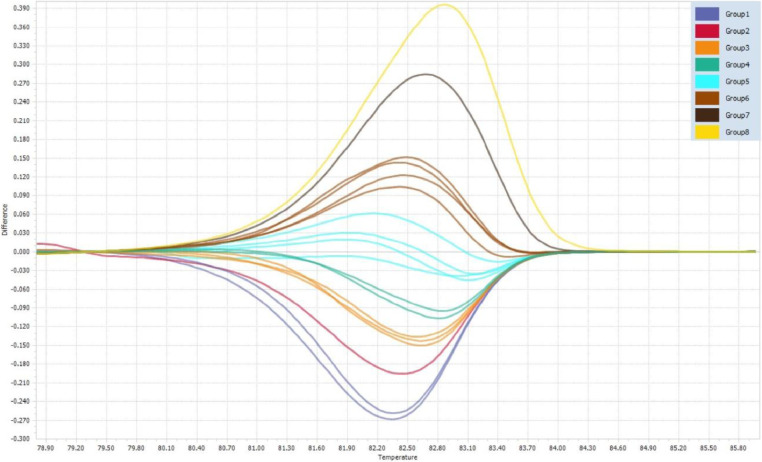
Difference plot of *C. parvum* subtype groupings.

This method successfully differentiated the studied subtypes into 8 subtype groupings, as specified in Table 6 and highlighted in Fig. 1. This method also has the potential to be expanded upon to include other tandem repeat loci for improved *C. parvum* subtype discrimination, adopting a multi-locus genotyping approach that is commonly employed in routine MLVA and population genetics analyses of *Cryptosporidium* species.

**Table 6:** Intra- and inter-assay reproducibility of melting temperatures of *C. parvum gp60* subtypes exhibited by HRM analysis.

gp60 subtype	Subtype grouping	Intra-assay Reproducibility				Inter-assay Reproducibility			
		T_m_* 1	T_m_* 2	Mean T_m_*±SD⁎⁎	%CV⁎⁎⁎	T_m_* 1	T_m_* 2	Mean T_m_*±SD⁎⁎	%CV⁎⁎⁎
IIaA21G1R1	1	82.02	81.96	81.99±0.04	0.052	81.94	81.88	81.91±0.04	0.052
IIaA20G1R1	1	82.02	82.02	82.02±0.00	0.000	82	81.94	81.97±0.04	0.052
IIaA16R1	2	82.16	82.22	82.19±0.04	0.052	82.14	82.08	82.11±0.04	0.052
IIaA20G2R1	3	82.29	82.29	82.29±0.00	0.000	82.21	82.21	82.21±0.00	0.000
IIaA17G1R1	3	82.24	82.29	82.27±0.04	0.043	82.34	82.27	82.31±0.05	0.060
IIaA18G1R1	3	82.29	82.35	82.32±0.04	0.052	82.28	82.21	82.25±0.05	0.060
IIaA16G1R1	4	82.42	82.42	82.42±0.00	0.000	82.4	82.41	82.41±0.01	0.010
IIaA15G1R2	4	82.48	82.42	82.45±0.04	0.051	82.41	82.34	82.38±0.05	0.060
IIaA17G2R1	5	82.55	82.62	82.59±0.05	0.060	82.6	82.54	82.57±0.04	0.051
IIaA21G3R1	5	82.49	82.55	82.52±0.04	0.051	82.4	82.4	82.40±0.00	0.000
IIaA20G3R1	5	82.55	82.55	82.55±0.00	0.000	82.54	82.47	82.51±0.05	0.060
IIaA19G3R1	5	82.62	82.68	82.65±0.04	0.051	82.54	82.47	82.51±0.05	0.060
IIaA15G2R1	6	82.81	82.8	82.81±0.01	0.010	82.8	82.73	82.77±0.05	0.060
IIaA18G3R1	6	82.81	82.81	82.81±0.00	0.000	82.73	82.67	82.70±0.04	0.051
IIaA17G3R1	6	82.75	82.75	82.75±0.00	0.000	82.79	82.73	82.76±0.04	0.051
IIaA19G4R1	6	82.81	82.88	82.85±0.05	0.060	82.8	82.8	82.80±0.00	0.000
IIaA17G4R1	7	83.14	83.14	83.14±0.00	0.000	83.12	83.06	83.09±0.04	0.051
IIaA10G2R1	8	83.32	83.29	83.31±0.02	0.025	83.26	83.26	83.26±0.00	0.000

⁎ Melting temperature.

⁎⁎ Standard deviation.

⁎⁎⁎ Coefficient of variance.

## Method validation

### Reproducibility of HRM analysis

In order to assess the intra-experimental reproducibility, duplicate reactions were analysed of the subtype samples within a single PCR run. Inter-experimental variation was assessed in separate, duplicate runs of identical subtype sample composition.

Based on the data obtained from intra- and inter-experimental reproducibility experiments, the averages, standard deviations, and coefficients of variation of T_m_ peaks generated by the HRM software were calculated and indicated high levels of reproducibility, as outlined in Table 6.

### Limit of detection evaluation

The limit of detection (LoD) for both *gp60* outer and inner primer pairs was evaluated using *Cryptosporidium* DNA extracted from a semi-purified (*via* salt floatation) faecal sample containing *Cryptosporidium parvum* oocysts. Prior to extraction, oocysts within a semi-purified faecal sample were microscopically enumerated in triplicate with KOVA Glasstic Slides (Medical Supply Company, Dublin, Ireland), with the average oocyst concentration determined to be approximately 1 × 10^4^ oocysts per ml. DNA was extracted from oocyst samples, following the same SPS extraction protocol outlined previously for clinical samples. A 10-fold serial dilution, ranging from 1 × 10^4^ oocysts per ml to 1 × 10^−2^ oocysts per ml was prepared from the *C. parvum* genomic extract.

**Fig. 2:**
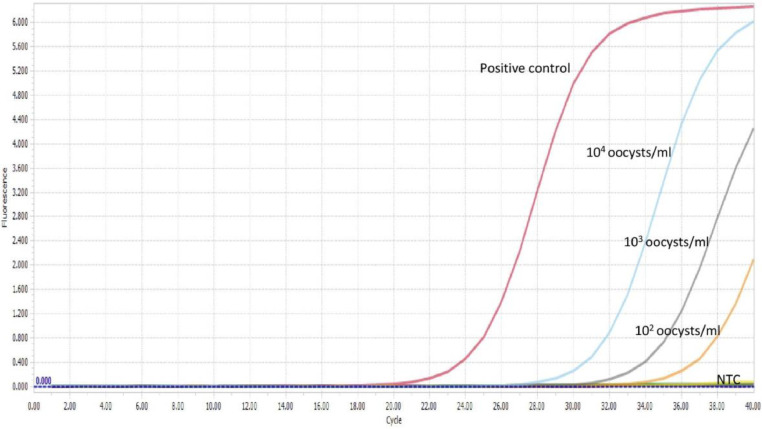
Real-time PCR amplification curves of *C. parvum* outer gp60 primer pair sensitivity evaluation.

**Fig. 3:**
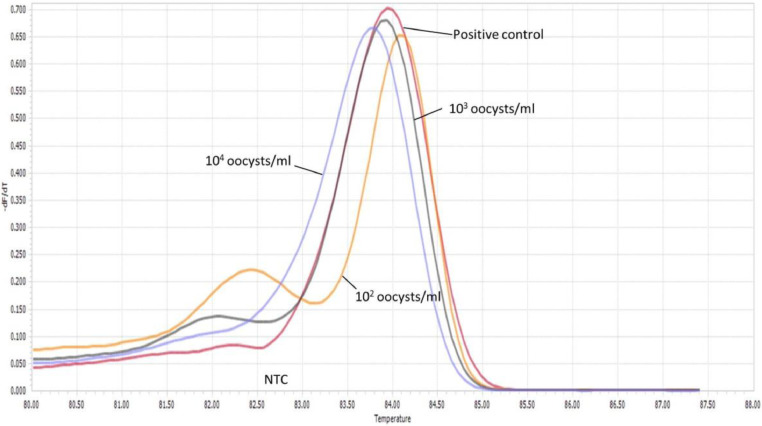
HRM analysis melting peaks of *C. parvum* outer primer pair sensitivity evaluation.

**Fig. 4:**
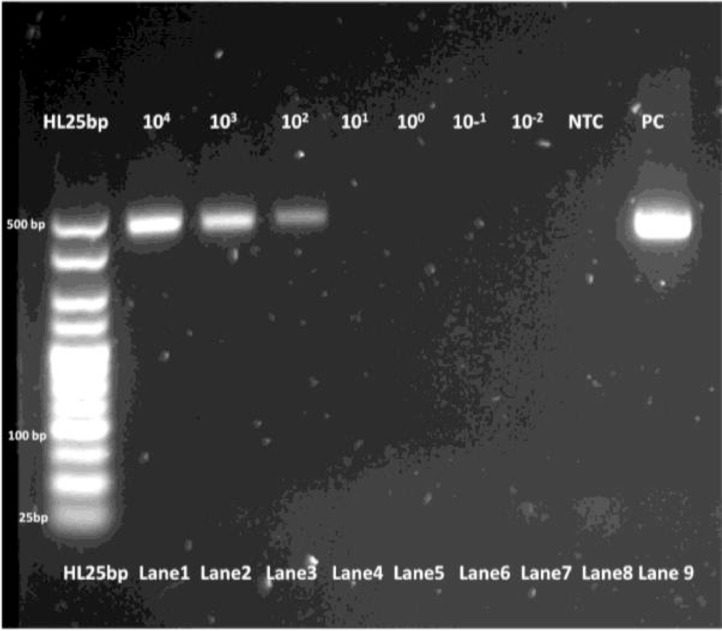
PCR products of *C. parvum* outer gp60 primer pair sensitivity evaluation visualized via gel electrophoresis.

**Fig. 5:**
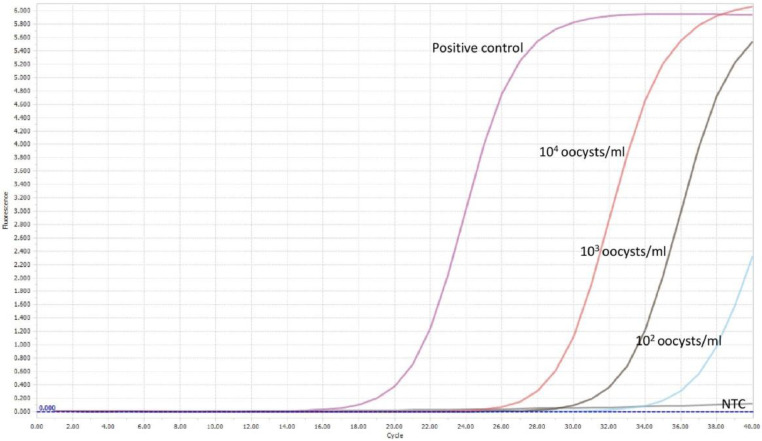
Real-time PCR amplification curves of *C. parvum* inner gp60 primer pair sensitivity evaluation.

**Fig. 6:**
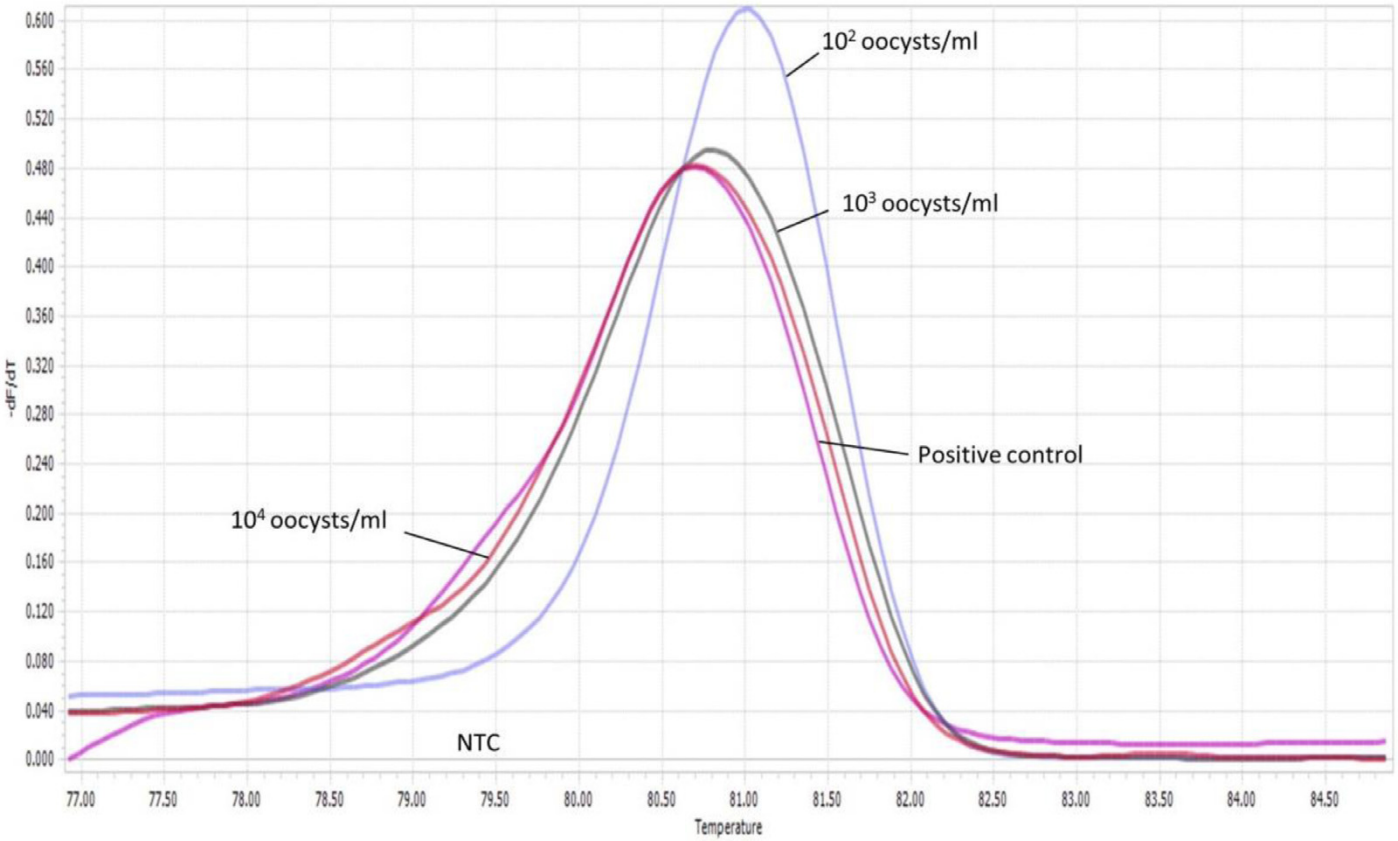
HRM analysis melting peaks of *C. parvum* inner primer pair sensitivity evaluation.

**Fig. 7:**
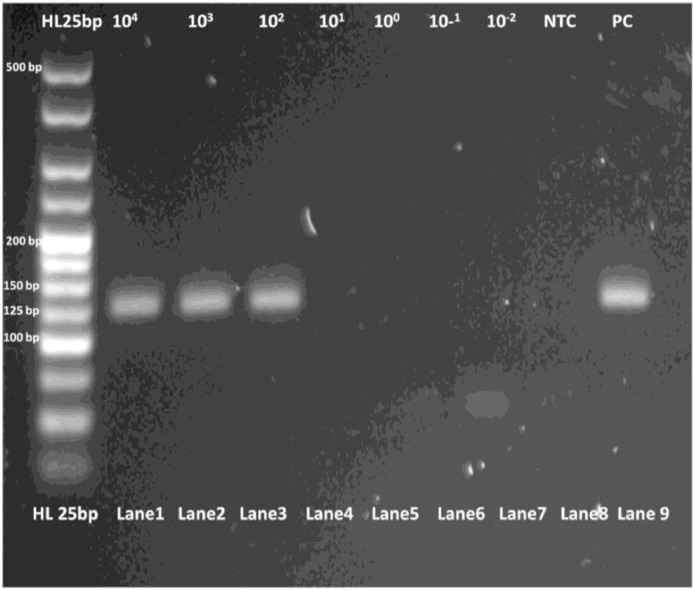
PCR products of *C. parvum* inner gp60 primer pair sensitivity evaluation visualized via gel electrophoresis.

The limit of detection for both *C. parvum gp60* outer and inner primer pairs was assessed employing the respective amplification conditions outlined previously, and gel electrophoresis. All reactions were conducted in duplicate. For clarity, single reactions are shown in Figs. 2, 3, 5 and 6. The limit of detection for both outer and inner primer pairs was 1 × 10^2^ oocysts/ml, as seen in Fig. 2, Fig. 3, Fig. 4, Fig. 5, Fig. 6, Fig. 7.

### Specificity evaluation

The specificity of both *C. parvum* outer and inner *gp60* primers sets was also evaluated, using DNA extracts from varying enteric pathogens, of various genera, both bacterial and parasitic in nature. *Salmonella, Shigella, Campylobacter*, Verotoxigenic *Escherichia coli* (VTEC) and *G. lamblia* positive faecal DNA extracts detected during routine molecular enteric screening in CUH were tested, in addition to genomic DNA extracted from *Blastocystis hominis* cysts (ATCC, United States of America). *Salmonella, Shigella, Campylobacter*, Verotoxigenic *Escherichia coli* (VTEC) and *G. lamblia* were extracted on-site in the Medical Microbiology Department of CUH, via the EntericBio SPS based protocol as outlined previously. DNA was extracted from *B. hominis* cysts employing the DNeasy Blood and Tissue Kit (Qiagen, German), as per manufacturer's instructions for DNA extraction from Gram-negative bacteria.

**Fig. 8:**
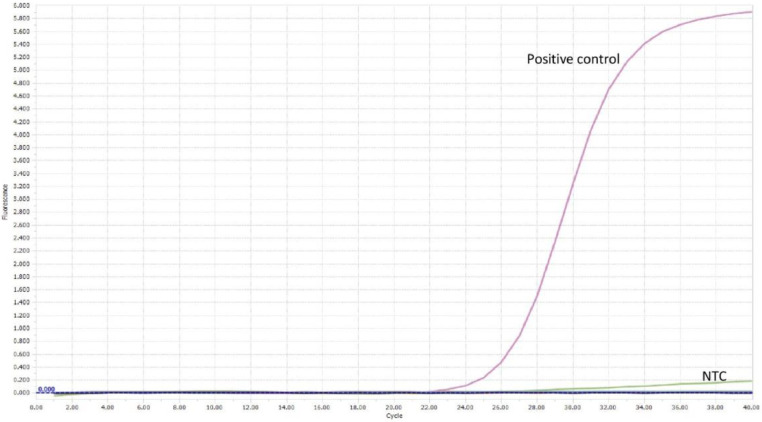
Real-time PCR amplification curves of *C. parvum* outer gp60 primer pair specificity evaluation.

**Fig. 9:**
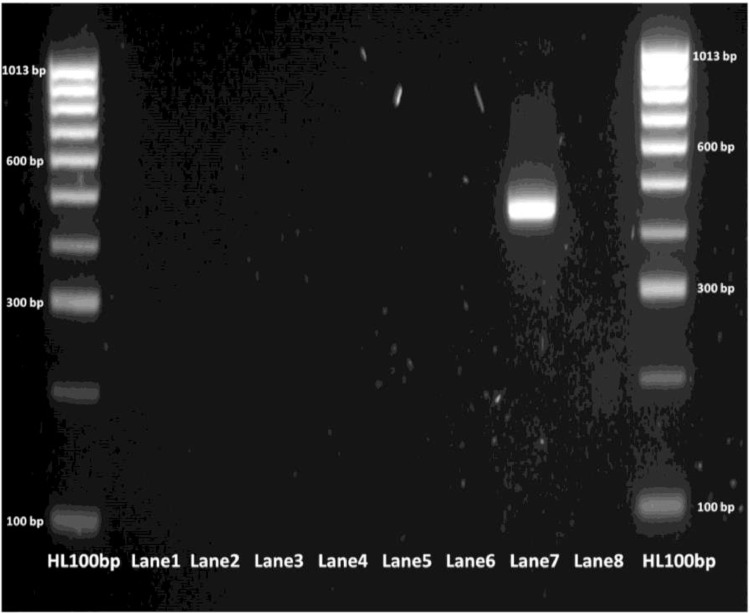
PCR products of *C. parvum* outer gp60 primer pair specificity evaluation visualized via gel electrophoresis. Lane 1 - *Salmonella.*; Lane 2 - *Shigella*; Lane 3 - *Campylobacter*; Lane 4 - VTEC; Lane 5 - *G. lamblia*; Lane 6 - *B. hominis*; Lane 7 - *C. parvum* (positive control); Lane 8 - NTC.

**Fig. 10:**
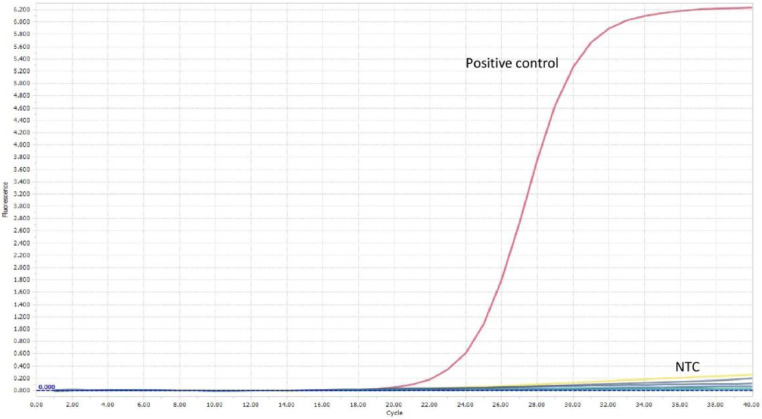
Real-time PCR amplification curves of *C. parvum* inner gp60 primer pair specificity evaluation.

**Fig. 11:**
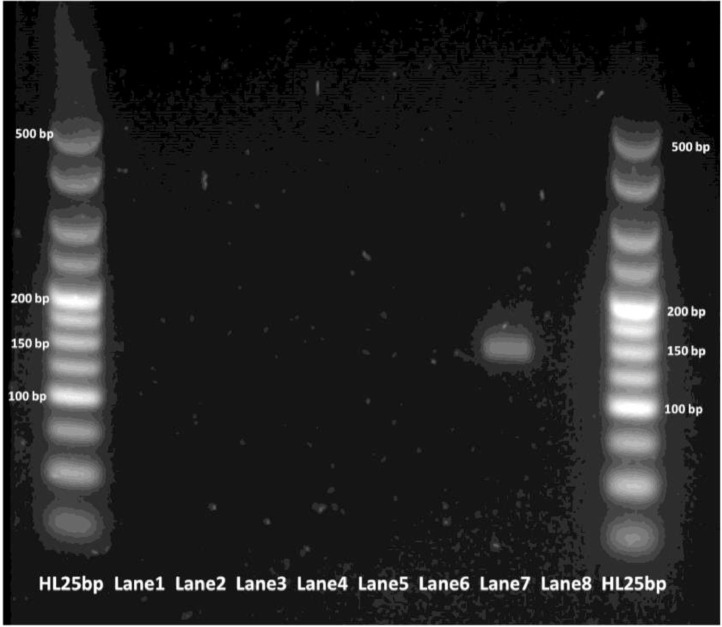
PCR products of *C. parvum* inner gp60 primer pair sensitivity evaluation visualized via gel electrophoresis. Lane 1 - *Salmonella.*; Lane 2 - *Shigella*; Lane 3 - *Campylobacter*; Lane 4 - VTEC; Lane 5 - *G. lamblia*; Lane 6 - *B. hominis*; Lane 7 - *C. parvum* (positive control); Lane 8 - NTC.

Extracted DNA was amplified under the real-time PCR conditions highlighted previously for both *gp60* primer sets, respectively. All reactions were conducted in duplicate. Amplification of the *C. parvum* template DNA alone was observed for both primer pairs, as seen in Fig. 8, Fig. 9, Fig. 10, Fig. 11.

## Declaration of Competing Interest

There are no conflicts of interest, of which we are aware, relating to this body of work.
